# Driving Simulator Training Is Associated with Reduced Inhibitory Workload in Older Drivers

**DOI:** 10.3390/geriatrics1030016

**Published:** 2016-07-04

**Authors:** Gianclaudio Casutt, Mike Martin, Lutz Jäncke

**Affiliations:** 1 Department of Psychology, Division of Neuropsychology, University of Zurich, Binzmühlestrasse 14/25, CH-8050 Zurich, Switzerland; 2 Department of Psychology, Division of Gerontopsychology, University of Zurich, Binzmühlestrasse 14/24, CH-8050 Zurich, Switzerland; 3 International Normal Aging and Plasticity Research Centre (INAPIC), CH–8050 Zurich, Switzerland; 4 Center for Gerontology, CH-8050 Zurich, Switzerland; 5 University Research Priority Program “Dynamics of Healthy Aging”, University of Zurich, CH-8050 Zurich, Switzerland; mike.martin@uzh.ch

**Keywords:** cognitive training, brain plasticity, aging, driving skills, driving simulator, inhibition process

## Abstract

Background: In demanding cognitive tasks, older people mostly experience more problems than younger people, and their brain workload is higher. An overloaded or exhausted mental workload is frequently associated with unsafe driving behavior. In this paper, we hypothesize that 10 active training sessions in a driving simulator positively influence brain workload, which relates to a beneficial increase in on-road driving performance. Methods: Ninety-one healthy active drivers (62–87 years) were randomly assigned to: (a) a driving simulator-training group; (b) an attention-training group; or (c) a control group. The dependent variables of this training study were brain workload (theta Fz/alpha Pz), and performance in three tasks, for which inhibition of inadequate responses (Stroop, Negative Priming, and Flanker) is required. Seventy-seven participants (85% of the total sample) completed the training. Training gains were analyzed by using a multiple regression analysis with planned comparisons. Results: The results revealed that the driving simulator training reduced brain workload during performance of the inhibition tasks. The performance of the simulator group during the inhibition tasks did not improve, but the participants completed the tasks with less brain workload compared to the attention-training group. Conclusion: Adding to our first paper on the Drive-Wise project, this paper now focuses on the superiority of the driving simulator training, compared to attention-training in regards to reducing brain workload. The change in brain workload seems to be associated with a positive change in drivers’ behavior on the road. Hence, a driving simulator training lasting only ten sessions leads to beneficial neuroplastic changes. This demonstrates brain plasticity of older people and its possible positive influence in real driving behavior.

## 1. Introduction

Driving a car is a demanding task [1] that is known to be problematic for a subset of older drivers due to declines in different cognitive domains [2]. Therefore, different countries have implemented specific policies to identify unsafe older drivers, and if necessary, to revoke a driving license [3]. Nevertheless, it has been shown that several of these policies have mostly failed [4,5]. However, driving practice interventions, especially for older drivers, may be an alternative approach to counteract reduced driving performance.

Focusing on driving in different age groups, many studies have demonstrated that the type of car accident differs between young and old drivers [6], and that there is an increased risk for older drivers to be involved in collisions, in more complex traffic situations [7,8]. Similarly, in complex traffic situations, older drivers tend to make more driving errors than younger drivers [9]. Anstey and Wood [2] reported on complex traffic situations, in which cognitive domains, such as selective attention, switching, inhibition, and discrimination predicts braking/accelerating and lane change errors in older drivers.

These differences between younger and older drivers are also discussed in the context of changes in cognitive decline, and driving safety [10,11], unsafe driving behavior in older drivers [12], and the use of compensatory behavior [13,14]. While educational approaches have been shown to raise awareness for problematic driving aspects in the older driver community [15], they did not increase driving safety in this age group [16]. Based on this knowledge, driving practice interventions may be an alternative approach to counteract reduced driving performance. In recent years, different training approaches for older drivers have been developed. For example, cognitive training studies have revealed promising training effects on driving safety [17], a delay in driving cessation [18], or the enhancement of protective driving behavior [9,19]. Roenker and colleagues [20] revealed that a speed of processing training compared to a passive video-watching in a driving simulator cabin increased driving performance. In addition, practicing visual scanning at intersections (second look) in a driving simulator leads to positive long-term effects, measured two years afterwards (number of second look) [21]. The reason for the advantages of interactive driving simulator training is likely due to the complexity of engaging multiple cognitive abilities within a comparable environment to real life. This is in accordance with previous research, which has demonstrated cognitive training benefits for older subjects, when practicing complex and strategic computer games (e.g., improved executive functions) [22].

In this paper, we will use the mental workload concept as a theoretical concept explaining older drivers’ ability to drive a car safely and efficiently. Mental workload is a frequently used concept in the context of traffic research, and particularly in research disciplines examining the efficiency of drivers’ performance. O’Donnel and Eggemeier [23] define mental workload as “the portion of an individual’s limited mental capacity that is actually required by task demands”. As has been mentioned by several authors, mental workload “emerges from the interaction between the requirements of a driving task, the circumstances under which it is performed, and the skills, behaviors, and perceptions of the pilot” or driver [24,25]. In this context, “situational awareness” is also an important concept. Borghini et al. [24] define situational awareness as the capability of drivers for the “perception of different environmental elements, with respect to time and space, together with a comprehension of their meaning, and the projection of their status after some variable has changed with time.” Mental workload and situational awareness are tightly connected as an increase in mental workload (induced by increased task demands), which most probably leads to a decrease in situational awareness, which in turn will result in decreased task performance. From the above-mentioned definitions, it is clear that very skilled drivers can manage more demanding traffic situations effectively, and with less errors. Thus, improving driving skills will reduce mental workload, and in turn will improve driving performance. The still unanswered question is, which kind of trainings might improve driving and cognitive skills in older and younger drivers.

In the context of aging, mental workload might increase faster and be sustained for a longer time, in more or less normal and average traffic situations, in a subset of older subjects. The reasons for this could be a general age-dependent cognitive decline, less driving skills due to less driving practice, or an unnecessary anticipation of driving problems caused by a negative self-image of the older driver. The latter possible reason is most likely evident in countries where older subjects are explicitly targeted as traffic risks. Therefore, increasing the mental workload capacities of older subjects might have beneficial effects on the driving performance in these drivers. Possible interventions that could be employed to increase mental workload, in the context of driving, could be intensive driving trainings (increase of driving skills), intensive cognitive trainings (increase of cognitive capacities), or optimization of the self-concept to a more self-confident, and realistic view of their own driving performance.

A study that focused on different training methods for older people (single vs. multitasking training) has been recently published by Anguera et al. [26]. The results of these training approaches (tracking or discrimination task vs. combination of the two tasks), demonstrated stronger changes in behavioral and neural measures in participants conducting multitasking training, compared to single task training. Better task performance (reaction time) and higher frontal theta power were observed in the multitasking training group [26]. Thus far, it seems that multitasking training approaches show greater benefits or stronger transfer effects to different higher order cognitive domains [27,28]. In order to drive correctly, more than one perceptual and cognitive process is needed [29]. The effective simultaneous usage of several cognitive processes during driving (e.g., multitasking) declines with chronological age and affects the level of mental workload [30]. Mastering traffic situations that are demanding seems to be a form of multitasking, for which the mental workload increases [31,32,33]. For example, whenever a dual-task exercise is necessary, the mental workload increases, whereas the overall driving performance decreases [34].

There are some validation studies for driving simulator applications. A high association (*r* = 0.716) or model fit (*R*^2^ = 0.657), in a sample of 129 older drivers, between virtual and real on-road driving behavior was found [35]. In addition, another study demonstrated that driving simulator behavior correlated with on-road driving performance (*r* = 0.599) and cognitive performance (*r* = 0.474), or a good model fit (*R*^2^ = 0.50) in older participants [36]. Furthermore, Engstrom and colleagues [32] have revealed a relationship between mental workload in real and simulator driving. As shown, it is possible to use driving simulators as interactive training instruments for traffic relevant aspects in cognition and/or behavior [9,19]. One of the advantages of a driving simulator is the possibility to train multiple cognitive processes, which are needed during active driving in real time, in a controlled laboratory setting.

Cantin and colleagues [37] compared younger and older participants in their handling of a multitude of different complex traffic situations in a simulator. For both groups, the mental workload increased with the complexity of the driving context. Nevertheless, younger drivers responded more than twice as often to an additional task, drove faster than older drivers, and in general showed a lower mental workload in a complex driving context. Therefore, the authors concluded that in situations requiring a higher mental workload, older drivers use compensation strategies (e.g., slower driving) that lower the mental workload. Furthermore, Bélanger et al. [29] revealed that older drivers who crashed in simulated overtaking maneuvers reported more “feelings of mental workload” (subjective distress) compared to non-crashers. Additionally, their cognitive test performance was lower in different cognitive domains (processing speed and attention). Correct inhibition and fast discrimination seem to be key processes for better short-time decision making, which is essential for driving. As in the study by Anstey and Wood [2], reaction time as a dependent variable is not sensitive enough to evaluate driving performance.

For correct and safe traffic navigation, it is of utmost importance that particular mental processes are still operative to a particular degree. To drive a car efficiently, several psychological functions need to be orchestrated. Most prominent of which are executive functions, including inhibition, attention, planning, and working memory (WM). However, it has frequently been argued that, due to old age, inhibitory processes can be impaired, which has led to the inhibitory deficit theory of aging [38,39,40]. In this theory, inhibition is considered to be a mechanism that suppresses ongoing or competing psychological functions. People with poor inhibitory abilities have difficulties with attention control, as well as with memory function [41], and additionally, with driving a car [42,43]. As inhibition capabilities strongly depend on the amount of workload [44], older people may demonstrate less inhibition when the workload increases. Strategies or trainings adapted to improve inhibition performance could be efficient in lowering the workload and consequently, enhancing driving performance in old age.

A promising neurophysiological measure for mental workload, which currently has only been used in the context of studies examining younger subjects, is the EEG based *brain workload* score [45,46,47]. This brain workload score is defined as the ratio between frontal theta and parietal alpha power (theta Fz/alpha Pz), as measured from standard EEG registrations [46,48,49]. Increased task demands, and therefore, enhanced mental workload, is also associated with an increased frontal theta activity and a simultaneous decrease in parietal alpha activity [46,48,49,50]. Several studies have used this brain workload score during driving situations. Thus far, they have demonstrated that this score changes during cognitive tasks that require more mental workload, and because of increased traffic demands [51,52]. Extensive demands on executive functions concern frontoparietal EEG coherence in the alpha and theta bands [50], and are associated with an increased brain workload score [49]. In a recent study, brain activity was recorded during driving in a simulator, which involved tasks of varying difficulty (with or without alertness and vigilance tasks). The results showed that the brain workload increased in relation to the level of task difficulty [51]. Lei and Roetting [52] observed in a younger sample, different mental workloads (WM: n0, n1 and n2 back) and driving conditions (passive/active, perform lane change during 75 or 100 km/h). During task execution, brain activity was recorded, which revealed that lane change deviation, WM error rate, and response time delay increased with driving task load and WM load. Moreover, brain workload increased with WM load.

In summary, evidence in the literature on mental workload, in the context of driving behavior, suggests that multitasking influences brain activity in older adults [26], and that the probability of driving errors increases simultaneously with increases in mental workload [37]. Therefore, brain workload might also relate to driving performance in older drivers. In our first paper from the Drive-Wise project at the University of Zurich, we found a greater benefit in on-road driving for older drivers participating in driving simulator training, compared with an attention-training group. The attention and the driving simulator training groups were both associated with general cognitive improvements, but only the participants from the driving simulator training group improved their on-road driving performance after training [53].

In this paper, we will report a second finding, obtained from this project. Here we report changed brain workload measures as a consequence of the different training regimes. All participants performed three inhibition tasks, before and after training while EEG brain workload measures were obtained. These psychological tests and the EEG measures took place in the same weeks as the on-road test drives and application of the cognitive test battery. Thus, we obtained performance measures of the inhibition tasks and brain workload measures, before and after the training interventions.

We hypothesize that the driving simulator training will result in more efficient processing of several psychological functions, including alertness, selective attention, as well as inhibition of irrelevant processes. All these processes are highly relevant for effectively and safely driving a car. Since the subjects who practiced driving a car will improve their attention and inhibition skills, brain workload will also substantially diminish when they perform attention and inhibition tasks outside the driving simulator. Thus, we anticipate a transfer from the driving simulator training to the performance in typical executive function tasks. Such kinds of transfer will not be present, or to a much smaller degree, for the cognitive training or the control subjects.

## 2. Materials and Methods

### 2.1. General Study Design

**Figure 1:**
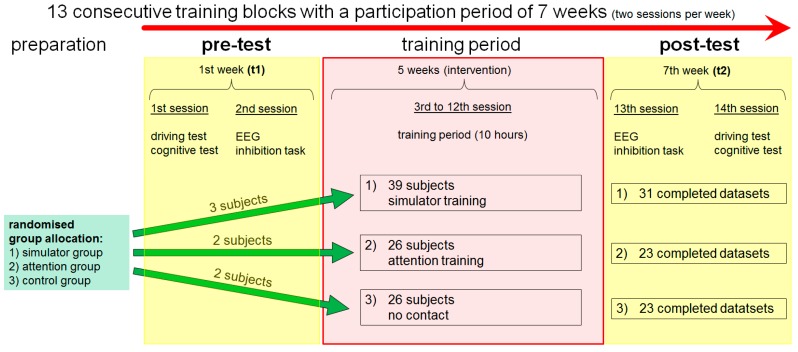
Study design.

As shown in Figure 1, our study was designed as a pre–post study with three independent groups. Participants were enrolled into one of two different training interventions (Group 1: Simulator training, Group 2: Attention training), or into a waiting group (Group 3: Control group). Due to ethical aspects, the waiting group received the simulator training after the post-test. The reason for the assignment of the subjects to the three groups (3:2:2) was a postulated increased dropout rate, due to simulator-sickness. Data acquisition took place during a period of 25 months (May 2010–June 2012). During the course of the entire experiment, all subjects came to the laboratory for 14 sessions in total, with Sessions 1 and 2 being conducted before the training period. The training period was comprised of 10 sessions (Sessions 3 to 12, with five weeks between Sessions 3 and 12) and the post-test period comprised two further sessions (Sessions 13 and 14). During Sessions 1 and 14, all subjects conducted an on-road driving test and a cognitive test battery. In the 2nd and 13th session, the subjects worked on tasks measuring specific executive functions (inhibition tasks: Stroop, Priming, and Flanker,) during which EEG was recorded. The results, which we obtained during sessions 1 and 14, as well as the general results of the training interventions, have been reported in one of our group’s previous papers [53]. This paper summarizes that the driving simulator training is associated with improved on-road driving performance, and also with improved performance in the cognitive test battery. In the current paper, we focus on the brain workload measure obtained from the EEG recording during performance of the inhibition tasks in the 2nd and 13th session. In addition, we will also examine the influence of the training interventions on performance during the inhibition tasks.

### 2.2. Training Interventions

**Figure 2:**
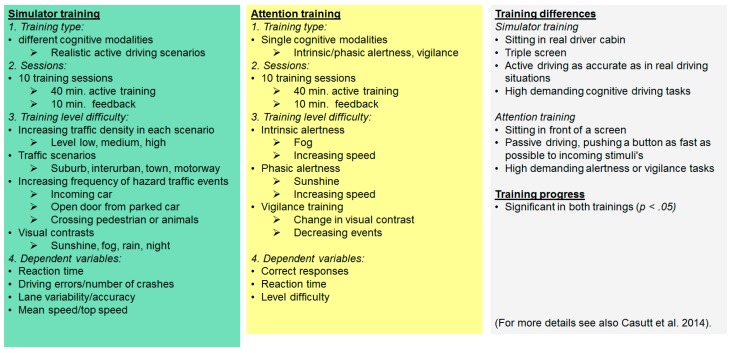
Characteristics of the different trainings, and their most prominent differences.

A schematic overview over the training interventions is shown in Figure 1. In addition, the most important differences between the driving simulator and attention training are shown in Figure 2.

*Driving simulator training:* In the driving simulator training, realistic driving scenarios, with increasing complexity, traffic density, and difficulty (hazardous events) were used. Participants drove actively in a realistic driver’s cabin (simulator-type: Trainer F12PT-1L, software version 12, Dr. Foerst GmbH, Dr.-Ing. Reiner Foerst, Wiehl, Germany) during the driving scenarios, and were instructed to drive as accurately as possible. We have described the driving simulator training in detail in our previous paper, thus we only briefly present the main issues here. A training session involved approximately 40 min of active driving through different scenarios, including interurban, suburban, town, and motorway, plus 10 min of feedback on reaction time, number of errors, and driving skills.

*Attention training:* It was of the same length as the driving simulator training. The training was comprised of practicing intrinsic (fog as a visualization for reducing visual contrast), and phasic (sunshine as a visualization for better visual contrast) alertness, as well as practicing vigilance (see also [54]). All attention trainings were conducted in front of a 15ʺ computer screen. The response panel and other hardware were products of Schuhfried GmbH [55]. Participants in the attention training group completed three training tasks consecutively (intrinsic and phasic alertness as well as vigilance), in each session. The software automatically increased the task difficulty when the subjects completed less demanding task levels without any errors (software provided by Schuhfried GmbH). For intrinsic and phasic alertness trainings, participants drove passively on a virtual motorcycle, and had to react by pushing a button whenever an object appeared in their lane. These were cognitive trainings, simulating traffic situations on a computer screen rather than being in the driving simulator. By introducing foggy traffic situations, or by increasing the speed of the virtual motorcycle, the demands of these training tasks were increased. With increasing speed, the reaction time decreased for a correct response (pushing a corresponding button). During vigilance training, participants drove passively in a virtual car and when an overtaking car’s backlight was shining, participants had to push a corresponding button. Task difficulty increased with a decrease of events.

### 2.3. Participants

We included in our analysis, those participants who qualified for the drive-wise project (the Zurich Drive-Wise project [53]). Therefore, we only briefly report the sample demographics. All participants had to be at least 60 years old and were recruited by newspaper advertisements. They received a briefing about the project and completed different questionnaires about traffic relevant aspects of their health (exclusion criteria: psychiatric, neurological or driving relevant illness, orthopedic issues, medication influencing driving and sensory impairment) and driving behavior. Based on this information the subjects were selected for this experiment. In Switzerland, drivers older than 70 years have to conduct a biennial medical check to test their driving relevant fitness, a public guideline that has been defined by the national traffic department. All participants had a valid driver’s license and were randomly allocated to one of the three training groups, already mentioned above: the simulator training group (*n* = 39), the attention training group (*n* = 26), and the control group (*n* = 26). The entire sample was comprised of 77 participants, with a mean age of 72.36 ± 5.61 (range 62–87), and included 55 men (71.4%). The different sample sizes of the three groups are due to different dropout rates in the three groups (primarily due to simulator sickness). The annual driving distance per year ranged between 8973 and 11,909 km. During the training period, the two training groups had to fill out a motivational and an emotional questionnaire. No group differences, with respect to demographic variables (age, level of education, gender), health status (neurological, psychiatric disorder, orthopedic problems, visual acuity) or driving behavior (active driving status, years of possession of driving license, exposition of driving context), were observed. We instructed the subjects not to practice car driving during the time of study participation, but although the subjects indicated that they did not practice, we had no opportunity to objectively test whether they had done so. For more details, see also Casutt et al. [53].

### 2.4. EEG Recording and EEG Data Analysis

EEG was recorded during the inhibition tasks (see below). EEG and eye blinks were recorded with a QuickAmp amplifier (Brain Products GmbH, Gilching, Germany). Sixty-four EEG and two eye channels (vertical and horizontal eye movements) were collected simultaneously during the experiments with a sampling rate of 500 Hz, recorded with a 16 bit A/D converter and BrainVision Recorder software (Brain Products GmbH). All electrodes were referenced to a common average reference, and the impedances were maintained lower than 30 kΩ. Brain activity was recorded with Ag/AgCl impedance-optimized electrodes (ActiCap, Brain Products GmbH Gilching, Germany). Active electrodes were placed according to the 10–20 system, and were fixed into an EasyCap with an adhesive patch. The ground electrode was positioned at AFz. The artifact-free and band pass filtered (0.1–30 Hz) EEG data were segmented to 2 s epochs, starting 0.5 s before and ending 1.5 s after stimulus presentation. These epochs were obtained for each task and for all correct answered trials separately for every participant. For every 2-s segment, Fast Fourier transforms (FFT) were calculated, which were then averaged to produce mean power spectra [45]. The sweeps were smoothed using a Hanning window (window length 10% without compression; resolution: 0.488 Hz). Absolute power spectra for frontal theta (4–8 Hz) and parietal alpha (8–12 Hz) were calculated for electrodes Fz (for theta) and Pz (for alpha) and exported for statistical analysis. These power values were processed to a frequently used brain workload score, by computing the ratio between frontal theta (theta Fz) and parietal alpha (alpha Pz) (TAR = (theta Fz)/(alpha Pz)). After this preprocessing, more than 200 power values for the theta and alpha band were obtained for every participant and task. Here we used the theta/alpha ratio as a measure for brain workload [49]. BrainVision Recorder and Presentation software (Neurobehavioral Systems, Albany, CA, USA) were used to manage EEG data recording, task presentation, and synchronization between EEG recording and task presentation. All inhibition tests conducted during EEG recording were presented and controlled via a Windows computer. The monitor (17-inch flat screen) presenting the task stimuli was placed in the Faraday shielded cage, in which the EEG recordings were conducted.

### 2.5. Inhibition Tasks

Three inhibition tasks were used for this experiment: Stroop, Negative Priming and Flanker. The dependent variables for these tasks were: reaction time (RT) and error rates (ER).

The *Stroop task* [56,57] was adapted from Hanslmayr*,* et al. [58], as a test stimulating deliberate inhibition. In this test, participants were required to suppress word reading. It consisted of eight blocks of two different conditions: four blocks of congruent, and four blocks of incongruent trials (each block contained 36 trials). Blocks occurred in alternating order, and the starting block changed after each measure. Stimuli were presented on a black screen in random order. In the congruent condition, the German words ‘‘ROT’’ (red), ‘‘GRÜN’’ (green), “GELB” (yellow) and ‘‘BLAU’’ (blue) were presented on the screen in their respective color. In the incongruent condition the four words were presented in one of the other three incongruent colors (e.g., ‘‘BLAU’’ in color yellow, green, or red). All possible combinations of word-color pairs were presented with equal probability. Participants were instructed to push the corresponding button of the words’ color as fast and as accurate as possible. The maximal presentation time was three seconds. Between trials there was a fixation cross with random time interval between 1 to 1.5 s. RT and errors were registered in log files. The Stroop effect was observed: *t*-test comparison before training sessions (T1) showed significantly faster RT, and fewer errors in the congruent trials (905 vs. 1078 ms, *t(*76) = 10.4, *p* < 0.01; 2.3 vs. 5.6 errors, *t*(76) = 4.5, *p* < 0.01).

The *Negative Priming (NP) task* was adapted from Andrés, et al. [59], as an unaware inhibition process. On a white screen, two overlapping capital letters, one in green and the other in red, were presented for 500 ms. One letter was always a vowel and the other a consonant (vowels A, E, O, U; consonants H, K, N, R). Participants were instructed to decide if the red letter was a vowel or a consonant by pushing a corresponding button (e.g., left button for vowels), as fast and as accurately as possible. The task consisted of four blocks of two different conditions: two blocks of NP and two blocks containing no NP (each block contained 56 trials). Blocks occurred in alternating order and the starting block changed after each measure. NP was defined as the distractor (green letter) in trial *n* and was the target (red letter) in trial *n* + 1. All possible letter combinations of vowel and consonant pairs were presented with equal likelihood in a random order. After stimulus onset, a white screen was presented with a maximal presentation time between intervals from 2.3 to 2.5 s. Before the presentation of the next stimulus, a fixation cross with random time interval between 0.3 to 0.5 s was presented. RT and errors were registered in log files. The NP effect was observed: t-test comparison before training sessions (T1) showed significantly faster RT and fewer errors in trials without NP compared with NP (671 vs. 688 ms, *t*(76)=3.78, *p* < 0.01; 5.9 vs. 7.1 errors, *t*(76) = 2.3, *p* < 0.05).

The *Flanker task* [60] used in this study was adapted from Salthouse [61], as a task requiring a deliberate inhibition process. Participants were required to suppress responses to salient stimuli. On a black screen one of four different arrow compositions was presented. Two stimuli were compatible (“‹‹‹‹‹” and “›››››”) and two stimuli were incompatible (“››‹››” and “‹‹›‹‹”). Participants were instructed to decide in each trial if the arrow in the middle (red only in this text) points to the left or the right by pushing a corresponding button (left button for left arrow pointing arrow, right button for right pointing arrow) as fast and as accurately as possible. The task consisted of four blocks, including both conditions in 72 trials: compatible and incompatible trials were presented with equal likelihood in random order. The maximal presentation time was three seconds, between trials a fixation cross was presented with random time intervals ranging from 1 to 1.5 s. RT and errors were registered in log files. In this task, the Flanker effect was clearly present: t-test comparison before training sessions (T1) showed significantly faster RT and fewer errors in compatible trials (622 vs. 835 ms, *t*(76) = 9.73, *p* < 0.01; 0.9 vs. 2.9 errors, *t*(76) = 4.5, *p* < 0.01). Reaction time differences between inhibition and neutral trials (RT_Inhibition_ minus RT_Neutral_) for the NP and Flanker tasks, as well as between incongruent and congruent trials (RT_Incongruent_ minus RT_Congruent_) for the Stroop task used as measures representing inhibition performance [58,59,61].

### 2.6. Statistical Analysis

Group differences were calculated at baseline (pre-test) using one-way ANOVAs for the performance in the inhibition tasks and brain workload. The following dependent variables were used to represent inhibition performance and brain workload: (1) reaction time differences between inhibition and non-inhibition trials, as well as error rates; and (2) frontal theta and parietal alpha power ratio (TAR), as a neurophysiological indicator of brain workload. As already mentioned in our previous paper, there were no baseline differences with respect to the performance in the cognitive test battery, on-road driving, or demographic variables. There were also no differences with respect to the inhibition performance, or the TAR brain workload measure between the three groups. As described in our first publication, a significant training progress for both training groups was detected. Additionally, there was no between-groups difference in emotional or motivational aspects [53].

To test training related changes with respect to the performance in the inhibitory tasks, and the brain workload measure, hierarchical multiple regression analysis, with planned group comparisons of the training benefits (pre–post differences for the dependent variables), was carried out. For the planned group comparisons, orthogonal contrast coding was used in accordance with the hypotheses formulated in the introduction.

On the basis of our hypothesis formulated in the introduction, two a-priori (planned) contrasts were designed, allowing us to examine potential interaction effects [62]. Therefore, we designed interaction contrasts allowing us to test pre–post differences between both training groups (attention and driving simulator training) and the control group. The second contrast was designed to compare the two training groups. For statistical testing we used a significance level of *p <* 0.05. We also report trends for statistical significance, which are defined as effects associated with a *p* > 0.05 and a *p* < 0.10. Since we used planned orthogonal contrasts, no further statistical significance testing was possible. Further comparisons were made using Cohen’s *d* effect size [63] measure to descriptively describe effects. Therefore, these effects can only be used to describe effects in this particular sample and for this particular experimental setup [64]. A *d*-value > 0.3 and <0.5 is considered as small, a *d*-value > 0.5 and <0.8 as moderate, while a *d*-value >0.8 is considered as large.

## 3. Results

The data from all 77 participants obtained during the inhibition tasks were included in the analysis. Due to technical problems during the EEG recording for one participant, this participant was excluded from the final analysis. Thus, the statistical analysis reported here relies on 76 participants.

Training gains on behavioral data for all three paradigms (Stroop, Negative Priming, Flanker) revealed no significant differences between the training groups and the control group, or between the simulator training group and the attention-training group. Descriptive statistics from pre and post inhibition performances are displayed in Table 1. Compared to the control group, there was no significant linear improvement as a consequence of training, with respect to the reaction time difference in the Stroop task (*F*(1,74) = 0.46, *p* = 0.50), in the Negative Priming task (*F*(1,74) = 0.03, *p* = 0.86), and the Flanker task (*F*(1,74) = 0.39, *p* = 0.53). Planned contrasts between simulator training and attention training revealed no differences in reaction time difference (incongruent minus congruent trials) in the Stroop task (*F*(1,74) = 0.30, *p* = 0.59), in the Negative Priming (negative minus normal trial) task (*F*(1,74) = 0.44, *p* = 0.51), and the Flanker task (incompatible minus compatible trial) (*F*(1,74) = 0.02, *p* = 0.90). Due to these non-significant findings for the behavioral data, and the obvious similarity of the measures obtained during the pre- and post-tests, no effect sizes were calculated (all *d* values are approximately 0).

**Figure 3:**
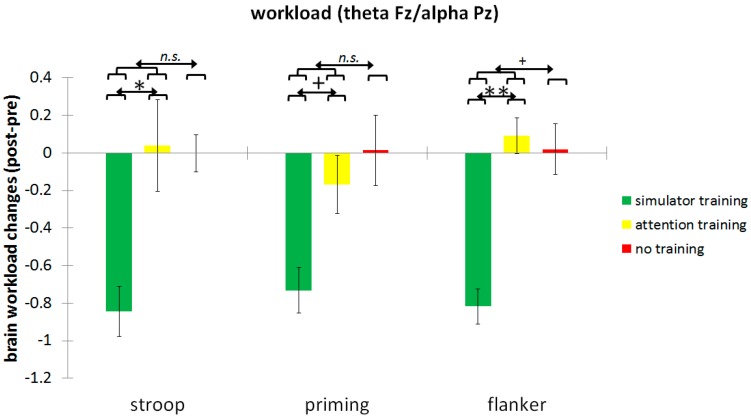
Change in the brain workload measures (theta Fz/alpha Pz), obtained during the inhibition tasks, and broken down for the three training groups (simulator training, attention training, no training). Note: Error bars in plots indicate the standard error of the mean; n.s. = not significant, **: *p* < 0.01; *: *p* < 0.05; +: *p* < 0.10.

Descriptive statistics of the brain workload changes from pre- and post-training conditions are displayed in Table 1, including Cohen’s *d* for the pre–post differences for the three groups. Interactions between the two specific contrasts, and the linear trend of training gains due to brain workload changes in each inhibition task are displayed in Table 2 and in Figure 3. Compared to the control group, there was no significant linear improvement in brain workload (*F*(1,73) = 0.88, *p* = 0.18) for the Stroop task as a result of the training, but there was a significant linear improvement for the simulator training group compared to the attention training group (*F*(1,73) = 3.49, *p* < 0.05). Compared to the control group, there was no significant linear improvement in brain workload (*F*(1,73) = 1.40, *p* = 0.12) for the Priming task as a result of the training, but a trend for linear improvement in the simulator training group, compared to the attention training group (*F*(1,73) = 1.68, *p* = 0.10). Compared to the control group, there was a trend for linear improvement in brain workload (*F*(1,73) = 2.28, *p* = 0.068) for the Flanker task as a result of the training, and a significant linear improvement in the simulator training group compared to the attention training group (*F*(1,73) = 5.86, *p* < 0.01).

## 4. Discussion

The main goal of this research was to investigate whether the performance in inhibition tasks and brain workload (indexed by theta Fz/alpha Pz) change because of different training regimes. In this paper, we examined the influence of a driving simulator and attention training, on inhibition tasks, performance, and brain workload. Supplementing our already published results on the benefits of driving simulator training [53], this paper demonstrates that realistic driving simulator training also reduces brain workload during performance of the inhibition tasks outside the driving simulator situation. No changes in the behavioral performance (inhibition task) was found, which emphasizes that fewer brain resources are needed for the same reaction time and error rates. The lack of change in the inhibition task performance can be explained as a consequence of the inhibition task paradigms, since no change in mental workload was included. For this reason, task difficulty did not change. In line with the neural changes in the driving simulator group, the task execution was easier after training than before. Since driving simulator training can be considered as multi-domain training, complex multi-domain trainings tend to exert greater efficacy than single-domain training approaches, in regards to various outcome measures [26,66]. In this context, it has also been shown that driving simulator training, as a model for multi-domain training, induces improvements in on-road driving [9,33], while single-domain trainings are less effective [22,27,28]. It is important to note that these improvements are associated with a shift in brain workload measures.

Recent publications have demonstrated the importance of complex training approaches, and their relationship to changes in mental and/or brain workload [26,66]. Our main finding is that older participants who took part in the simulator training, showed a decrease in brain workload during the performance of tasks requiring inhibitory functions. However, it is important to note that the performance in these tasks did not change, so the simulator training reveals an advantage over the other training regimes, with respect to the fact that less neuronal resources are needed after training, to conduct inhibitory tasks.

Further research could focus on the reasons for this selective influence of the simulator training. Our results support the idea that the driving simulator training requires more brain workload than a consecutive attention training, which has been proven by other authors. Cantin and colleagues [37] have demonstrated that driving complexity is associated with mental workload in older adults. Also, Lei and Roetting [52] revealed in a younger sample that brain workload increases in line with mental workload. Handling a driving simulator during different demanding traffic situations entails the efficient usage of several psychological functions (including inhibition of inadequate responses), either sequentially or simultaneously [29,30]. While practicing this demanding task, the subjects may learn to allocate the neurophysiological resources more efficiently [67]. Thus, this finding is in line with previous research, demonstrating that demanding multi-domain trainings tend to have greater efficacy or impact than single-domain trainings [22,27,28]. Moreover, this neuronal shift could be the reason why simulator trainings induce positive behavioral changes in on-road driving [9,21,33], in healthy older subjects.

Although the brain workload measures obtained during the performance of the inhibition tasks decrease as a consequence of the driving simulator training, the performance in these tasks remain unchanged, even for the attention training. The reasons for the lack of training effects on behavioral measures of executive functions in older subjects need further investigation [68,69]. It could be that the underlying neurophysiological processes change as a consequence of task-relevant, age-dependent compensational strategies. This idea is supported by a recent finding of Wild-Wall, Falkenstein and Hohnsbein [43]. Although these authors did not conduct a training study, they reported a type of dissociation between behavioral and neurophysiological indices of executive functions. They described a general slowing in performing executive tasks for the older subjects. This was not accompanied by reductions in interference effects, similar to those found in younger subjects. As the authors identified enhanced frontal N1 ERP amplitudes in older subjects, they suggested that older participants pay more attention to the task without influencing the task performance. Such compensatory neurophysiological activations are frequently found in studies comparing young and old subjects [70,71]. They are the pivotal argument for the posterior-anterior shift theory in aging [71].

Furthermore, it is important to note that the three inhibition tasks are not identical in relation to their theoretical framework. In the Stroop task, participants have to suppress reading. Therefore, participants must consciously suppress an automated brain function [72]. The Negative Priming task measures another automatic inhibitory process in addition to awareness [59]. In the current study, none of the participants realized the existence of a distractor, and as a result the Negative Priming effect was observed. The Flanker task requires non-automated brain functions, associated with inhibitory functions, of which the subjects are aware. The task difficulty is based on the fact that the subjects are required to actively suppress salient information with high task relevance [73]. Nevertheless, there is one common parameter in all three tasks, which is processing speed. Processing speed is frequently described as a typical function, for which older participants demonstrate weaker performance [74]. This “slowing” in older subjects is often evident in situations when different cognitive functions compete for control resources, which typically occurs in inhibition tasks [75]. Several studies have reported that older subjects use various compensatory brain resources to cope with these demanding situations [76,77,78,79]. Another study investigating the inhibitory deficit theory, found a difference in effect between younger and older adults, but no age-related interference effect [80]. The authors concluded that younger and older adults did not differ in their relative incapability to prevent the processing of irrelevant information. Transforming these findings to our results, we interpret the neuronal changes without any accompanying behavioral change, as a shift in cognitive strategies. Both training approaches included speed-sensitive components (feedback performance of reaction time), but at the behavioral level, no reduction in reaction time difference (RT_Inhibition_ minus RT_Neutral_) was observed. The change in brain workload in the simulator group may represent a shift from compensational, to more efficiently operating inhibitory processes, which needs less mental and neurophysiological resources. Anguera et al. [26] found comparable results and interpreted their findings as: *“training-induced neuroplasticity as the mechanistic basis of these training effects”*. Fronto-striatal circuits [81] play a crucial role, not only for motor functions, but also for cognitive and emotional functions as well. Tisch et al. [81] concluded the following on page 770.

“To summarize, of the fronto-striatal circuits described to date, only two, the motor and oculomotor circuits, have primarily motor functions. The remaining circuits are nonmotor and play a role in specific aspects of cognition or in regulation of drive, motivation, mood, and elements of social behavior.”

Based on the current results, some of these circuits might be used during the simulator training more extensively than in the single domain cognition training, due to multitasking, task novelty and complexity. In accordance with Gevins et al. [47], participants from the simulator training group completed inhibition tasks with equal performance, but needed less attention (reduced frontal theta), and were in a state of relaxation (increased parietal alpha) during task completion. Participants from the simulator group might perform with more flexibility and use less compensatory strategies after training [76,77]. In line with the current results, it seems that there are inhibitory fronto-striatal circuits with on-road driving relevance. Further research in this field should focus on these circuits and its influence on ageing and driving performance.

Our hypothesis was that the simulator training would induce psychological and neurophysiological processes, reducing the task-related brain workload level more than single attention trainings. Our hypothesis is grounded by the idea that complex driving simulator training, activates and trains several psychological functions, while consecutive single attention training generally makes use of only one psychological function. Therefore, when using a higher brain workload level for a longer time, the involved neural networks may have the opportunity to adapt to these demands, and develop a more efficient wiring, which at the end causes reduced brain workload levels during demanding tasks in general. In fact, we identified reduced brain workload levels during inhibitory tasks, only for those participants practicing the driving simulator. Further research needs to investigate how these gains affect driving safety, and whether complex training approaches can be implemented as tools for high-risk drivers, or for neurological rehabilitation programs.

## 5. Limitations

Since we used a priori defined contrasts, we could not test all possible training induced differences statistically. Therefore, we were only in the position to describe some potentially interesting training related differences descriptively.

A major limitation of this type of study is that it is unclear how long the observed beneficial effects of the driving simulator training will persist. There are some papers reporting reduced numbers of collisions [17], improved driving behavior, or cognitive performance for quite a long period after training [17,21,82]. However, further research is needed to examine the long-term consequences of driving simulator training.

Here we have used a relatively simple brain workload measure (theta Fz/alpha Pz), however, several other brain workload measures have been proposed, most likely reflecting more precisely the neurophysiological resources used, to control particular psychological functions [24,67,83,84,85].

It should also be noted that driving simulator trainings are also associated with some disadvantages. A fraction of the subjects suffers from simulator sickness or dizziness. In our study, we tested this explicitly, and excluded affected subjects, who suffered seriously from these disadvantageous states. However, we adapted our simulator setting in order to reduce possible simulator sickness feeling, by using smaller monitors and we disassembled the movable platform underneath the simulator seat that simulated car movements.

In addition, we did not control what the subjects did during the training period, when they were outside of our laboratory. Therefore, it might be that those subjects who had been enrolled in the driving simulator training were those who actually drove more in their car, because they had become more confident in their driving skills. It could also be possible that the specific training scenario stimulated other kinds of behavior, which the subjects perform more frequently outside the laboratory, which might be beneficial for the subjects. However, this is a general problem for almost all long-term training studies published so far, because it is rather difficult to control what the subjects actually do when they are outside the laboratory. Future studies using mobile devices might help to solve this problem.

Finally, we would like to acknowledge that we discussed our findings in the context of cognitive inhibitory control mechanisms, which were improved during the driving simulator training. The multiple factors (e.g., emotion, motivation, or for example personality) and its complexity is discussed in the review published by Nigg [86]. In our study, we controlled emotional and motivational aspects in our sample [53] and therefore we were not able to focus on the whole complexity of inhibitory control mechanisms. However, to formulate elaborate hypotheses about possible further influences on involved processes, in the context of inhibitory processes, we would need a different experimental design to delineate these different processes. Hopefully, future studies might be conducted accordingly.

## 6. Conclusions

In our first paper on the Drive-Wise project, we were able to demonstrate that driving simulator training improves on-road driving performance [53]. In the current paper, we elaborate the potential of driving simulator trainings to induce brain plasticity in older drivers. Active and complex simulator training, associated with high mental workload, reduces brain workload during inhibition tasks. In summary, this driving-simulator training induces a kind of optimization of inhibitory control. The trained participants exert inhibitory control with less neurophysiological “effort”. Consequently, this demonstrates that driving simulators are not only useful devices for improving on-road driving performance, but also for complex cognitive functions training. Further research needs to investigate how these gains affect driving safety (e.g., reduction in crash number), and whether complex training approaches can be implemented as tools for high risk drivers, or for neurological rehabilitation programs.

## Figures and Tables

**Table 1:** Descriptive statistics from all inhibition tasks and brain workload.

Performance Measure	Variable	Simulator Training Group (# *N* = 31; * *N =* 30)		*d*	Attention Training Group (*N* = 23)		*d*	Control Group (*N* = 23)		*d*
		Pre-Test	Post-Test		Pre-Test	Post-Test		Pre-Test	Post-Test	
		M (*SD*)	M (*SD*)		M (*SD*)	M (*SD*)		M (*SD*)	M (*SD*)	
Inhibition performance ^a^	Stroop: RT congruent trials in s	0.899 (0.130)	0.853 (0.147)		0.926 (0.124)	0.850 (0.131)		0.893 (0.130)	0.864 (0.118)	
	Stroop: RT incongruent trials in s	1.060 (0.193)	0.969 (0.184)		1.101 (0.199)	0.995 (0.179)		1.079 (0.203)	1.028 (0.172)	
	Priming: RT normal trials in s	0.660 (0.077)	0.648 (0.094)		0.664 (0.052)	0.651 (0.076)		0.696 (0.106)	0681 (0.089)	
	Priming: RT neg. prime trials in s	0.675 (0.080)	0.668 (0.089)		0.683 (0.055)	0.668 (0.079)		0.710 (0.090)	0.698 (0.083)	
	Flanker: RT compatible trials in s	0.626 (0.130)	0.586 (0.112)		0.612 (0.090)	0.593 (0.104)		0.623 (0.067)	0.591 (0.066)	
	Flanker: RT incomp. trials in s	0.825 (0.280)	0.722 (0.190)		0.834 (0.264)	0.751 (0.230)		0.849 (0.222)	0.737 (0.106)	
Brain workload	Stroop	2.06 (1.41)	1.34 (0.67)	0.54	2.03 (1.65)	2.06 (1.52)	0.01	1.41 (0.73)	1.40 (0.98)	0.01
	Priming	2.01 (1.23)	1.27 (0.57)	0.61	1.61 (1.28)	1.44 (0.70)	0.15	1.57 (1.71)	1.58 (1.00)	0.01
	Flanker	1.96 (1.03)	1.14 (0.80)	0.67	1.36 (0.84)	1.31 (0.86)	0.05	1.43 (1.28)	1.43 (1.00)	0.00

Notes: *d* = Cohen’s *d* effect size [64] using the pooled *SD* for both conditions and correcting for dependence between means according to Morris and DeShon [65]. # for on-road and behavioral performance scores, * for EEG measures, ^a^ Smaller scores reflect better performance, RT = reaction time, s = seconds.

**Table 2:** Multiple regression analysis for the interaction between orthogonal contrasts and linear trend for the brain workload.

Variable	*B*	*SE*	β
Brain workload during Stroop task			
Linear interaction AB × C	−0.067	0.071	−0.075
Linear interaction A × B	−0.221	0.118	−0.150 *
Brain workload during Priming task			
Linear interaction AB × C	−0.077	0.065	−0.094
Linear interaction A × B	−0.140	0.108	−0.103 ^+^
Brain workload during Flanker task			
Linear interaction AB × C	−0.072	0.048	−0.101 ^+^
Linear interaction A × B	−0.193	0.080	−0.162 **

Notes: A = driving simulator group; B = attention training group; C = control group; AB × C = comparison of the average of the training effect for group A and B versus the training effect for group C; A × B = comparison of the training effect for group A versus the training effect for group B; ** *p* < 0.01; * *p* < 0.05; ^+^ *p* < 0.10; *SE* = standard error.

## References

[1] Summala H. (1996). Accident risk and driver behaviour. Saf. Sci..

[2] Anstey K.J., Wood J. (2011). Chronological age and age-related cognitive deficits are associated with an increase in multiple types of driving errors in late life. Neuropsychology.

[3] OECD (2001). Ageing and Transport: Mobility Needs and Safety Issues.

[4] Casutt G., Martin M., Jäncke L. (2013). Alterseffekte auf die fahrsicherheit bei schweizer kraftfahrern im jahr 2010. Z. Verkehrssicher..

[5] Siren A., Meng A. (2012). Cognitive screening of older drivers does not produce safety benefits. Accid. Anal. Prev..

[6] Zhang J., Fraser S., Lindsay J., Clarke K., Mao Y. (1998). Age-specific patterns of factors related to fatal motor vehicle traffic crashes: Focus on young and elderly drivers. Public Health.

[7] Braitman K.A., Kirley B.B., Ferguson S., Chaudhary N.K. (2007). Factors leading to older drivers' intersection crashes. Traffic Inj. Prev..

[8] Owsley C., Ball K., McGwin G., Sloane M.E., Roenker D.L., White M.F., Overley E.T. (1998). Visual processing impairment and risk of motor vehicle crash among older adults. J. Am. Med. Assoc..

[9] Romoser M.R., Fisher D.L. (2009). The effect of active versus passive training strategies on improving older drivers’ scanning in intersections. Hum. Factors Soc..

[10] Rizzo M., McGehee D.V., Dawson J.D., Anderson S.N. (2001). Simulated car crashes at intersections in drivers with alzheimer disease. Alzheimer Dis. Assoc. Disord..

[11] Anstey K.J., Wood J., Lord S., Walker J.G. (2005). Cognitive, sensory and physical factors enabling driving safety in older adults. Clin. Psycholo. Rev..

[12] Joanisse M., Gagnon S., Voloaca M. (2012). Overly cautious and dangerous: An empirical evidence of the older driver stereotypes. Accid. Anal. Prev..

[13] Ross L.A., Clay O.J., Edwards J.D., Ball K.K., Wadley V.G., Vance D.E., Cissell G.M., Roenker D.L., Joyce J.J. (2009). Do older drivers at-risk for crashes modify their driving over time?. J. Gerontol. B Psychol. Sci. Soc. Sci..

[14] Hakamies-Blomqvist L. (1994). Compensation in older drivers as reflected in their fatal accidents. Accid. Anal. Prev..

[15] Stalvey B.T., Owsley C. (2003). The development and efficacy of a theory-based educational curriculum to promote self-regulation among high-risk older drivers. Health Promot. Pract..

[16] Owsley C., McGwin G., Phillips J.M., McNeal S.F., Stalvey B.T. (2004). Impact of an educational program on the safety of high-risk, visually impaired, older drivers. Am. J. Prev. Med..

[17] Ball K., Edwards J.D., Ross L.A., McGwin G. (2011). Cognitive training decreases motor vehicle collision involvement of older drivers. J. Am. Geriatr. Soc..

[18] Edwards J.D., Lunsman M., Perkins M., Rebok G.W., Roth D.L. (2009). Driving cessation and health trajectories in older adults. J. Gerontol. A Biol. Sci. Med. Sci..

[19] Lavallière M., Simoneau M., Tremblay M., Laurendeau D., Teasdale N. (2012). Active training and driving-specific feedback improve older drivers’ visual search prior to lane changes. BMC Geriatr..

[20] Roenker D.L., Cissell G.M., Ball K.K., Wadley V.G., Edwards J.D. (2003). Speed-of-processing and driving simulator training result in improved driving performance. Hum. Factors Soc..

[21] Romoser M.R. (2013). The long-term effects of active training strategies on improving older drivers’ scanning in intersections: A two-year follow-up to romoser and fisher (2009). Hum. Factors Soc..

[22] Basak C., Boot W.R., Voss M.W., Kramer A.F. (2008). Can training in a real-time strategy video game attenuate cognitive decline in older adults?. Psychol. Aging.

[23] O’Donnel R.D., Eggemeier F.T., Kaufman B.K., Wiley T.J. (1986). Cognitive processes and performance. Handbook of Perception and Human Performance.

[24] Borghini G., Astolfi L., Vecchiato G., Mattia D., Babiloni F. (2014). Measuring neurophysiological signals in aircraft pilots and car drivers for the assessment of mental workload, fatigue and drowsiness. Neurosci. Biobehav. Rev..

[25] Paxion J., Galy E., Berthelon C. (2014). Mental workload and driving. Front. Psychol..

[26] Anguera J.A., Boccanfuso J., Rintoul J.L., Al-Hashimi O., Faraji F., Janowich J., Kong E., Larraburo Y., Rolle C., Johnston E. (2013). Video game training enhances cognitive control in older adults. Nature.

[27] Lustig C., Shah P., Seidler R., Reuter-Lorenz P.A. (2009). Aging, training, and the brain: A review and future directions. Neuropsychol. Rev..

[28] Zelinski E.M. (2009). Far transfer in cognitive training of older adults. Restor. Neurol. Neurosci..

[29] Bélanger A., Gagnon S., Yamin S. (2010). Capturing the serial nature of older drivers’ responses towards challenging events: A simulator study. Accid. Anal. Prev..

[30] Hakamies-Blomqvist L., Mynttinen S., Backman M., Mikkonen V. (1999). Age-related differences in driving: Are older drivers more serial?. Int. J. Behav. Dev..

[31] Arien C., Jongen E.M., Brijs K., Brijs T., Daniels S., Wets G. (2013). A simulator study on the impact of traffic calming measures in urban areas on driving behavior and workload. Accid. Anal. Prev..

[32] Engstrom J., Johansson E., Ostlund J. (2005). Effects of visual and cognitive load in real and simulated motorway driving. Transp. Res. F Traffic Psychol. Behav..

[33] Lavallière M., Laurendeau D., Simoneau M., Teasdale N. (2011). Changing lanes in a simulator: Effects of aging on the control of the vehicle and visual inspection of mirrors and blind spot. Traffic Inj. Prev..

[34] Recarte M.A., Nunes L.M. (2003). Mental workload while driving: Effects on visual search, discrimination, and decision making. J. Exp. Psychol. Appl..

[35] Lee H.C., Cameron D., Lee A.H. (2003). Assessing the driving performance of older adult drivers: On-road versus simulated driving. Accid. Anal. Prev..

[36] Casutt G., Martin M., Keller M., Jäncke L. (2014). The relation between performance in on-road driving, cognitive screening and driving simulator in older healthy drivers. Transp. Res. F Traffic Psychol. Behav..

[37] Cantin V., Lavalliere M., Simoneau M., Teasdale N. (2009). Mental workload when driving in a simulator: Effects of age and driving complexity. Accid. Anal. Prev..

[38] Radvansky G.A., Zacks R.T., Hasher L. (2005). Age and inhibition: The retrieval of situation models. J. Gerontol. B Psychol. Sci. Soc. Sci..

[39] Zacks R.T., Hasher L., Dagenbach D., Carr T.H. (1994). Directed Ignoring: Inhibitory Regulation of Working Memory. Inhibitory Processes in Attention, Memory, and Language.

[40] Lustig C., Hasher L., Zacks R.T., Gorfein D.S., MacLeod C.M. (2007). Inhibitory deficit theory: Recent developments in a “new view”. The Place of Inhibition in Cognition.

[41] Hasher L., Zacks R.T., Rahhal T.A. (1999). Timing, instructions, and inhibitory control: Some missing factors in the age and memory debate. Gerontology.

[42] Daigneault G., Joly P., Frigon J.Y. (2002). Executive functions in the evaluation of accident risk of older drivers. J. Clin. Exp. Neuropsychol..

[43] Wild-Wall N., Falkenstein M., Hohnsbein J. (2008). Flanker interference in young and older participants as reflected in event-related potentials. Brain Res..

[44] Chmielewski W.X., Muckschel M., Stock A.K., Beste C. (2015). The impact of mental workload on inhibitory control subprocesses. NeuroImage.

[45] Gevins A., Smith M.E. (2000). Neurophysiological measures of working memory and individual differences in cognitive ability and cognitive style. Cereb. Cortex.

[46] Gevins A., Smith M.E., Leong H., McEvoy L., Whitfield S., Du R., Rush G. (1998). Monitoring working memory load during computer-based tasks with EEG pattern recognition methods. Hum. Factors Soc..

[47] Gevins A., Smith M.E., McEvoy L., Yu D. (1997). High-resolution EEG mapping of cortical activation related to working memory: Effects of task difficulty, type of processing, and practice. Cereb. Cortex.

[48] Gevins A., Smith M.E., McEvoy L.K., Ilan A.B., Chan C.S., Jiang A., Sam-Vargas L., Abraham G. (2011). A cognitive and neurophysiological test of change from an individual’s baseline. Clin. Neurophysiol..

[49] Holm A., Lukander K., Korpela J., Sallinen M., Muller K.M. (2009). Estimating brain load from the EEG. Sci. World J..

[50] Ward L.M. (2003). Synchronous neural oscillations and cognitive processes. Trends Cogn. Sci..

[51] Borghini G., Vecchiato G., Toppi J., Astolfi L., Maglione A., Isabella R., Caltagirone C., Kong W., Wei D., Zhou Z. Assessment of mental fatigue during car driving by using high resolution EEG activity and neurophysiologic indices. Proceedings of the 2012 Annual International Conference of the IEEE Engineering in Medicine and Biology Society.

[52] Lei S., Roetting M. (2011). Influence of task combination on EEG spectrum modulation for driver workload estimation. Hum. Factors Soc..

[53] Casutt G., Theill N., Martin M., Keller M., Jäncke L. (2014). The drive-wise project: Driving simulator training increases real driving performance in healthy older drivers. Front. Aging Neurosci..

[54] Hauke J., Fimm B., Sturm W. (2011). Efficacy of alertness training in a case of brainstem encephalitis: Clinical and theoretical implications. Neuropsychol. Rehabil..

[55] Schuhfried Cogniplus. http://www.schuhfried.com/cogniplus-cps/the-training-programs.

[56] Stroop J.R. (1935). Studies of interference in serial verbal reactions. J. Exp. Psychol..

[57] MacLeod C.M. (1991). Half a century of research on the stroop effect: An integrative review. Psychol. Bull..

[58] Hanslmayr S., Pastotter B., Bauml K.H., Gruber S., Wimber M., Klimesch W. (2008). The electrophysiological dynamics of interference during the stroop task. J. Cogn. Neurosci..

[59] Andrés P., Guerrini C., Phillips L.H., Perfect T.J. (2008). Differential effects of aging on executive and automatic inhibition. Dev. Neuropsychol..

[60] Eriksen B.A., Eriksen C.W. (1974). Effects of noise letters upon identification of a target letter in a non-search task. Percep. Psychophys..

[61] Salthouse T.A. (2010). Is flanker-based inhibition related to age? Identifying specific influences of individual differences on neurocognitive variables. Brain Cogn..

[62] Pedhazur E.J. (1982). Multiple regrssion. Explanation and prediction. Behavioral Research.

[63] Krauth J. (1988). Distribution-Free Statistics. An Application-Oriented Approach.

[64] Cohen J. (1988). Statistical Power Analysis for the Behavioral Sciences.

[65] Morris S.B., DeShon R.P. (2002). Combining effect size estimates in meta-analysis with repeated measures and independent-groups designs. Psychol. Methods.

[66] Takeuchi H., Kawashima R. (2012). Effects of processing speed training on cognitive functions and neural systems. Rev. Neurosci..

[67] Shriram R., Sundhararajan M., Daimiwal N. (2014). EEG based cognitive workload assessment for maximum efficiency. IOSR J. Electron. Commun. Eng..

[68] Wild-Wall N., Falkenstein M., Gajewski P.D. (2012). Neural correlates of changes in a visual search task due to cognitive training in seniors. Neural. Plast..

[69] Dahlin E., Nyberg L., Backman L., Neely A.S. (2008). Plasticity of executive functioning in young and older adults: Immediate training gains, transfer, and long-term maintenance. Psychol. Aging.

[70] Reuter-Lorenz P.A., Cappell K.A. (2008). Neurocognitive aging and the compensation hypothesis. Curr. Dir. Psychol. Sci..

[71] Davis S.W., Dennis N.A., Daselaar S.M., Fleck M.S., Cabeza R. (2008). Que pasa? The posterior-anterior shift in aging. Cereb. Cortex.

[72] West R., Baylis G.C. (1998). Effects of increased response dominance and contextual disintegration on the stroop interference effect in older adults. Psychol. Aging.

[73] Zeef E.J., Sonke C.J., Kok A., Buiten M.M., Kenemans J.L. (1996). Perceptual factors affecting age-related differences in focused attention: Performance and psychophysiological analyses. Psychophysiology.

[74] Salthouse T.A. (1996). The processing-speed theory of adult age differences in cognition. Psychol. Rev..

[75] Rush B.K., Barch D.M., Braver T.S. (2006). Accounting for cognitive aging: Context processing, inhibition or processing speed?. Neuropsychol. Dev. Cogn. B Aging Neuropsychol. Cogn..

[76] Braver T.S., Paxton J.L., Locke H.S., Barch D.M. (2009). Flexible neural mechanisms of cognitive control within human prefrontal cortex. Proc. Nati. Acad. Sci. USA.

[77] Puccioni O., Vallesi A. (2012). Conflict resolution and adaptation in normal aging: The role of verbal intelligence and cognitive reserve. Psychol. Aging.

[78] Berlingeri M., Danelli L., Bottini G., Sberna M., Paulesu E. (2013). Reassessing the harold model: Is the hemispheric asymmetry reduction in older adults a special case of compensatory-related utilisation of neural circuits?. Exp. Brain Res..

[79] Reuter-Lorenz P. (2002). New visions of the aging mind and brain. Trends Cogn. Sci..

[80] Feyereisen P., Charlot V. (2008). Are there uniform age-related changes across tasks involving inhibitory control through access, deletion, and restraint functions? A preliminary investigation. Exp. Aging Res..

[81] Tisch S., Silberstein P., Limousin-Dowsey P., Jahanshahi M. (2004). The basal ganglia: Anatomy, physiology, and pharmacology. Psychiatr. Clin. N. Am..

[82] Edwards J.D., Myers C., Ross L.A., Roenker D.L., Cissell G.M., McLaughlin A.M., Ball K.K. (2009). The longitudinal impact of cognitive speed of processing training on driving mobility. Gerontologist.

[83] Berka C., Levendowski D.J., Lumicao M.N., Yau A., Davis G., Zivkovic V.T., Olmstead R.E., Tremoulet P.D., Craven P.L. (2007). EEG correlates of task engagement and mental workload in vigilance, learning, and memory tasks. Aviat. Space Environ. Med..

[84] Stikic M., Berka C., Levendowski D.J., Rubio R.F., Tan V., Korszen S., Barba D., Wurzer D. (2014). Modeling temporal sequences of cognitive state changes based on a combination of EEG-engagement, EEG-workload, and heart rate metrics. Front. Neurosci..

[85] Dijksterhuis C., de Waard D., Brookhuis K.A., Mulder B.L., de Jong R. (2013). Classifying visuomotor workload in a driving simulator using subject specific spatial brain patterns. Front. Neurosci..

[86] Nigg J.T. (2000). On inhibition/disinhibition in developmental psychopathology: Views from cognitive and personality psychology and a working inhibition taxonomy. Psychol. Bull..

